# Tuneable magnetic nanocomposites for remote self-healing

**DOI:** 10.1038/s41598-022-14135-8

**Published:** 2022-06-17

**Authors:** Ranjeetkumar Gupta, Priya Gupta, Charles Footer, Gavin B. G. Stenning, Jawwad A. Darr, Ketan Pancholi

**Affiliations:** 1 Advanced Materials Group, School of Engineering, Robert Gordon University, Aberdeen, AB10 7GE UK; 2 Lakshmi Narain College of Technology and Sciences, RGPV, Indore, MP India; 3 Department of Chemistry, University College London, 20 Gordon Street, London, WC1H OAJ UK; 4 ISIS Neutron and Muon Facility, STFC Rutherford Appleton Laboratory, Didcot, OX11 0QX UK

**Keywords:** Composites, Mechanical engineering, Synthesis and processing

## Abstract

When polymer composites containing magnetic nanoparticles (MNPs) are exposed to an alternating magnetic field, heat is generated to melt the surrounding polymer locally, partially filling voids across any cracks or deformities. Such materials are of interest for structural applications; however, structural polymers with high melting temperatures pose the challenge of generating high localised temperatures enabling self-healing. A method to prepare a multiferroic-Polyamide 6 (PA6) nanocomposite with tuneable magnetocaloric properties is reported. Tunability arises from varying the MNP material (and any coating, its dispersion, and agglomerate sizes in the nanocomposite). The superparamagnetic MNPs (SMNPs) and iron oxide MNPs with and without surface functionalization were dispersed into PA6 through in situ polymerization, and their magnetic properties were compared. Furthermore, computer simulations were used to quantify the dispersion state of MNPs and assess the influence of the interaction radius on the magnetic response of the self-healable magnetic nanoparticle polymer (SHMNP) composite. It was shown that maintaining the low interaction radius through the dispersion of the low coercivity MNPs could allow tuning of the bulk magnetocaloric properties of the resulting mesostructures. An in-situ polymerization method improved the dispersion and reduced the maximum interaction radius value from ca. 806 to 371 nm and increased the magnetic response for the silica-coated SMNP composite. This sample displayed ca. three orders of magnitude enhancement for magnetic saturation compared to the unfunctionalized Fe_3_O_4_ MNP composite.

## Introduction

Well-engineered magnetic nanocomposites with magnetocaloric capability are of interest in several engineering fields, including biomedical or structural self-healable magnetic nanoparticle polymer (SHMNP) composites^1–3^. Several groups worldwide are developing bulk polymer composites that can autonomously repair themselves through interaction with stimuli such as incident magnetic fields^4–6^. Furthermore, some of the authors recently reported the development of self-healing flexible composite pipelines using the magnetocaloric effect^7^. In that work^7^, a composite multilayer tape consisting of a low melting temperature SHMNP sandwiched between a high melting point thermoplastic unidirectional fibre-reinforced prepreg or tape formed the basis of the self-healing pipe. When this tape was exposed to microwave radiation, the constituent polymer was shown to melt due to the magnetocaloric effect of nanoparticles dispersed within the structure^7^. This was due to the formation of a liquified (melted) polymer that filled the microcracks in the composite pipe (made up of unidirectional fibre-reinforced prepreg), causing the composite to self-heal. In this space, the optimization of the magnetic properties of the magnetic tape is essential for successful energy-efficient self-healing.

Many parameters, such as the choice of magnetic material, size and composition of MNPs, polymer type, and dispersion of MNPs in the polymer matrix, contribute to tuning the magnetocaloric properties of the SHMNP composites. Furthermore, maximisation of the saturation magnetisation to coercivity ratio for any material increases magnetocaloric efficiency. Low magnetic coercivity with appropriate degree of crystallinity is important to increase magnetic heating efficiency^8^. For example, the cubic phase crystalline gadolinium oxide nanoparticles with a low Curie temperature and coercivity is an ideal material with low coercivity for use in these self-healing structures, but its cost is prohibitive for the majority of applications^9^.

In SHMNP composites, the high overall interfacial area offered by adding a small amount of MNPs with a high surface area-to-volume ratio enhances magnetic properties^10,11^. Though controlling the dispersion of the MNPs in the polymer matrix, it is essential to achieve desirable magnetocaloric properties. The interaction of local magnetic fields of neighbouring large agglomerates of MNPs has been shown to decrease the magnetocaloric effect in some polymer composites^3^. To reduce the agglomeration size of MNPs and increase dispersion, surface functionalization (coating) of particles can be used in SHMNP composites.

Herein, the authors present a specific method of preparing several SHMNP composites from surface-functionalised (coated) MNPs and polyamide 6 (PA6). To evaluate the effect of dispersion and magnetic material composition of MNPs on the magnetisation and thermomagnetic properties of the prepared SHMNP composite samples, four types of samples were prepared—two with 1 w/w% Fe_3_O_4_ MNPs and two with 1 w/w% superparamagnetic MNPs (SMNPs). Further details of the sample contents and their nomenclature can be found in Table 1. The resulting magnetic properties showed some dependency on the silica functionalization (coating) of MNPs and on the preparation technique of the SHMNP composites. Superparamagnetic MNPs^12^ (synthesized by University College London authors) may be considered promising candidates for achieving a low Curie temperature at lower cost than other potential MNP filler options. The improvements in the dispersion state of MNPs and their interparticle interactions were assessed, as well as their resulting magnetic multifunctionality.

**Table 1:** Details of the prepared samples.

SHMNP composites sample in text	SHMNP composites sample description	SCN (w/w %)	NPC (w/w %)
Sample A	PP	–	–
Sample B	Un-Fe_3_O_4_	0	1.0
Sample C	TEOS-Fe_3_O_4_	2.4	1.0
Sample D	Un-SMNP	0	1.0
Sample E	TEOS-SMNP	2.4	1.0

*PP* pristine polymer (PA6), *Un-Fe*_*3*_*O*_*4*_ uncoated Fe_3_O_4_, *TEOS-Fe*_*3*_*O*_*4*_ silica coated Fe_3_O_4_, *Un-SMNP* uncoated superparamagnetic MNP, *TEOS-SMNP* silica coated superparamagnetic MNP, *SCN* silica coating on MNPs, *NPC* MNP concentration in SHMNP composites.

## Results and discussions

Establishing the correlation between particle dispersion state, magnetic particle properties including crystallinity and polymer crystallinity is required for optimizing the properties of the manufactured SHMNP composites. Thus, the dispersion states for four different SHMNP composite samples (Table 1) were evaluated using transmission electron microscopy (TEM), X-ray diffraction (XRD) and small and wide-angle X-ray scattering (SAXS/WAXS) and compared with the pristine synthesized PA6 polymer. The average MNP size and agglomerate size distribution within the SHMNP composites were estimated from SAXS/WAXS data through implementation of the Guinier and Porod law^3^. The calculated average MNP/agglomerate size was then used to build a 3D model for visualization. Furthermore, the magnetic properties of the MNPs and their SHMNP composites at temperatures of 100 and 400 K, respectively, were measured using a superconducting quantum interference device (SQUID) detection system (Quantum Design MPMS 3 integrated SQUID). To estimate the magnetocaloric properties, field-cooled (FC) and zero-field-cooled (ZFC) magnetization curves for the SHMNPs were obtained at various magnetic field strengths. This information was then correlated with the dispersion state. All characterization methods are described in detail in Supplementary Data Section S1.

The magnetic properties of the SHMNP composites include the ability to respond to even a small magnetic field and be capable of generating a sufficiently high temperature to melt a structural polymer similar to PA6. The presence of MNPs in the SHMNP composite reduces the overall melting temperature (due to a drop in the degree of crystallinity due to MNPs); however, a small crystallite size was ensured via the SHMNP composite processing methodology.

### Chemical composition of the prepared PA6 SHMNP composite samples

**Figure 1:**
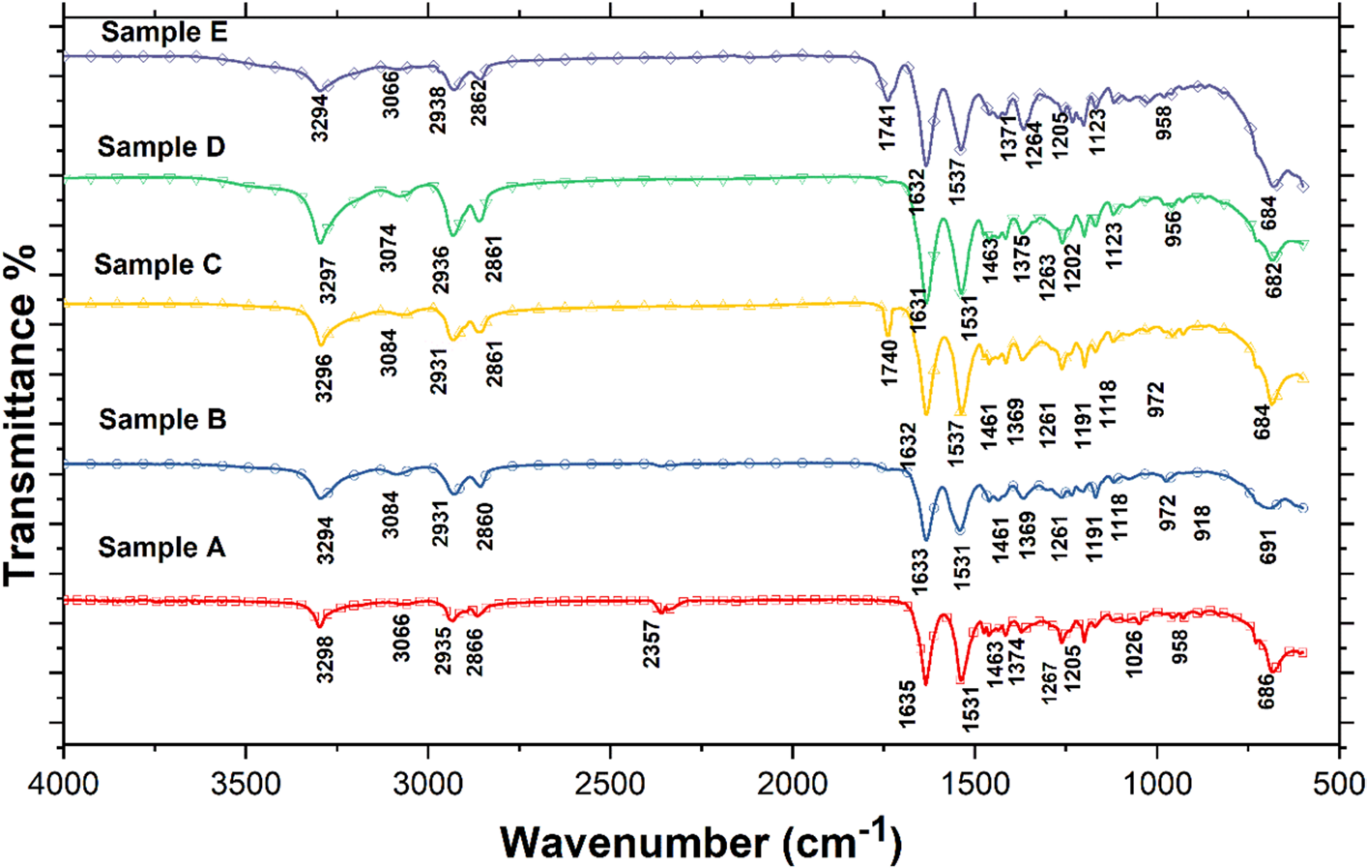
ATR-FTIR spectra of polymer and SHNMP composite samples.

Attenuated total reflectance-Fourier transform infrared (ATR-FTIR) spectral data for pure PA6 and all SHMNP (Fig. 1) composites were used to confirm the successful synthesis of PA6 by matching peaks associated with commercial grade PA6 reported in the literature^13^. As seen in Fig. 1, peaks related to methylene (CH_2_) asymmetric and symmetric stretching vibrations for the absorbance bands related to PA6 were observed in the ranges 2931 to 2938 cm^−1^ and 2860 to 2866 cm^−1^, confirming the formation of PA6^13,14^. Prominent absorbance bands corresponding to N–H stretching and hydrogen bonding were observed for the as-made and commercial samples in the range^3^ 3294 to 3298 cm^−1^. In addition, this absorbance band also confirmed the presence of the amide II band (of primary amides), which occurred due to stretching vibration of the C=O double bond, which can be further attributed to the functionality of the amide I band. Additional peaks were also seen ca. 1540 cm^−1^, relating to the previously mentioned primary type amide I and II bands but also the vibrations due to stretching of the C-N bond, which was accompanied by peaks due to bending of the N–H bond and the CO–NH bend^15^.

As seen in Fig. 1, the level of crystallinity of the SHMNP composite increased with MNP silica surface functionalization, which was confirmed by the observation of a shift in the N–H bending peaks from 1531 to 1537 cm^−1^ in samples C and E^14^. The degree of crystallinity was generally dictated by the position and intensity of these crystalline bands, as well as the broader bands from the amorphous phase^16^. It was inferred that the degree of crystallinity was moderately high in the SHMNP composite samples with the uncoated MNPs, as the amide II band (sensitive to crystallinity) appeared at approximately 1531 cm^−1^ in the spectra of samples B and D. The wavelength fingerprint for carboxylic acid (C=O stretch), a product from the hydrolysis of tetraethyl orthosilicate (TEOS) during the MNP coating process, is typically ca. 1700 cm^−1^, however, the closest corresponding absorbance band observed, was a small broad peak near the peak^17^ at ca. 1740 cm^−1^.

### Effect of silica coating on dispersion of nanoparticles

**Figure 2:**
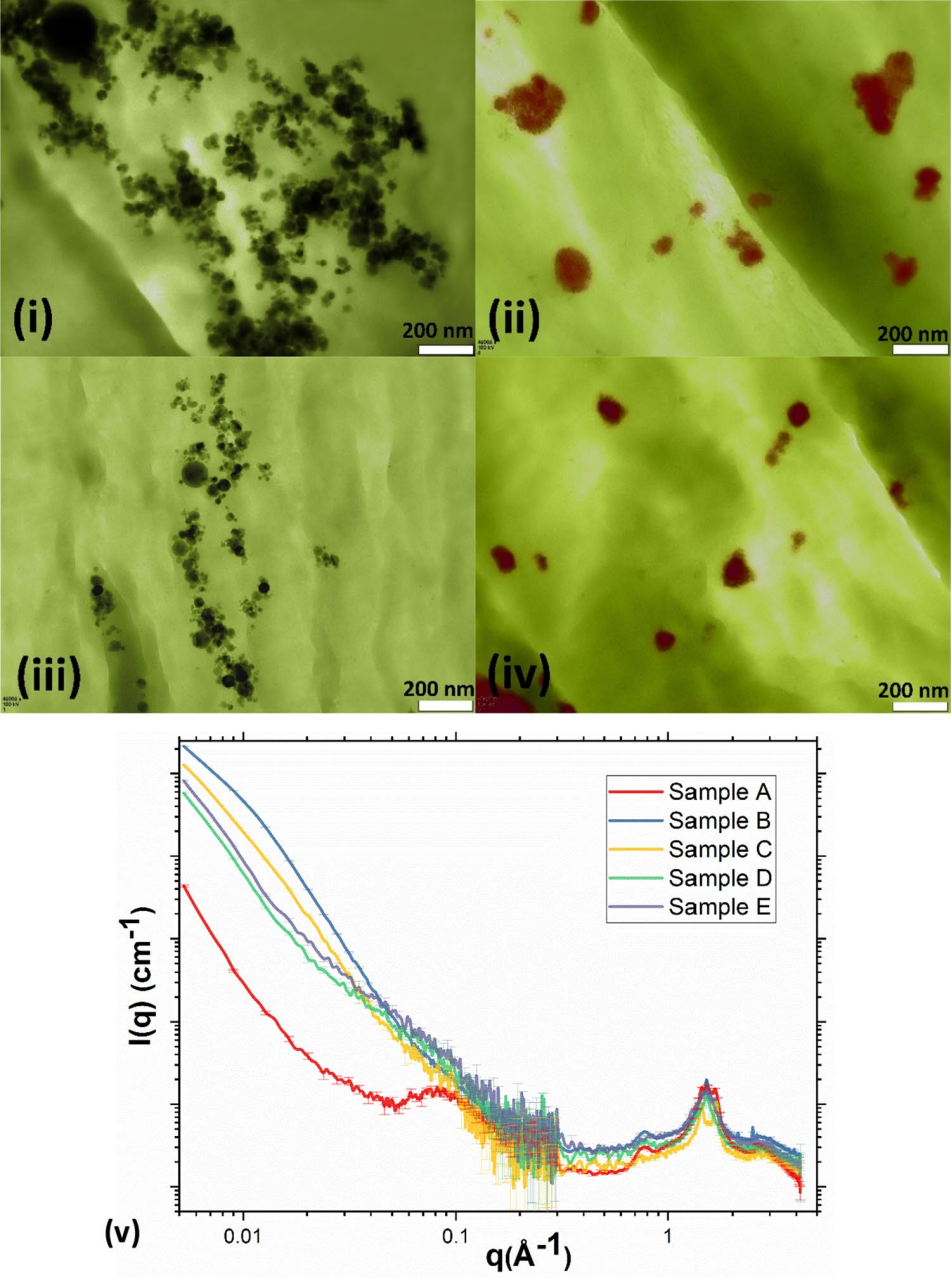
Postprocessed TEM images for microtome sections of nanocomposites containing uncoated samples (**i**) sample B and (**ii**) sample D and nanocomposites containing coated samples, (**iii**) sample C and (**iv**) sample E. (The scale bar shown is 200 nm.) The Fe_3_O_4_ and superparamagnetic MNPs are observed as black and red colours, respectively. (**v**) Background corrected SAXS (0.005–0.3 Å^−1^) and WAXS (0.3–4.17 Å^−1^) intensities I(q) as a function of the scattering vector ‘q’ for all the samples studied.

A good dispersion state of MNPs within SHMNP composites ensures a high polymer crystallinity, small crystallite size and uniform magnetic saturation, which is essential for efficient self-healing and mechanically strong joints. In the preparation of SHMNP composites, the MNPs were functionalized (coated with silica before being added to the PA6 matrix) to improve their dispersion state. To quantify the improved MNP dispersion state due to silica coating, the TEM images of SHMNP composites were analysed. The TEM images of the microtome SHMNP composite slices showed that the dispersion of MNPs (or their agglomerates) was improved with MNP silica coatings (see Fig. 2iii,iv). As seen in Fig. 2i,ii, the uncoated MNPs in samples B and D formed larger agglomerates due to high dipole–dipole interparticle attractions.

The TEM images only provided information on the dispersion state of the MNPs in an area of a few square micrometers. Therefore, SAXS/WAXS data were employed to help evaluate the dispersion state on a broader scale (microvolume) than TEM images. To determine the extent of dispersion, the SHMNP composites were characterized using SAXS/WAXS techniques. The filler structure and structure of polymer chains inside MNP-polymer composites (similar to SHMNP composites) have been previously studied with scattering techniques, including XRD and SAXS/WAXS^18^. Statistical mechanical theories relate the dispersion state (which dictates the space configuration of the nanoinclusions) to stress within the polymer^19^, and this nanoscale stress correlates to the size of inclusions/agglomerates. This helped elucidate MNP sizes within the SHMNP composite, including for complex agglomerates of MNPs^20^.

Figure 2v represents the scattering intensity I(q), plotted as a function of the scattering vector q for all the samples studied. The SAXS profile of the Guinier-type plot (Supplementary Data Section S2) was comprised of a flat region due to the polymer response (as is very clear from the pristine PA6 data plot) and a region with a steep gradient related to the response from the MNPs in the samples. The cumulatively slope-dropping region is a characteristic of the Porod scattering response from MNPs^21^. The Guinier region precedes the Porod region, wherein the scattering of the former reflects the radius of gyration of the scatterers as per Guinier’s law^21^. By calculating the radius of gyration (R_g_), the average diameter (D) of MNPs in a volume of SHMNP composites was estimated. These data are summarised in Table S1, while a detailed analysis of the SAXS data is discussed in Supplementary Data Section S2.

The average diameter (D) of MNPs calculated from the SAXS data was found to be in broad agreement with the values obtained from TEM image analysis. Samples C and E have the lowest average diameter of MNP/agglomerates.

### Effect of MNP dispersion on the crystallinity of SHMNP composite samples

**Figure 3:**
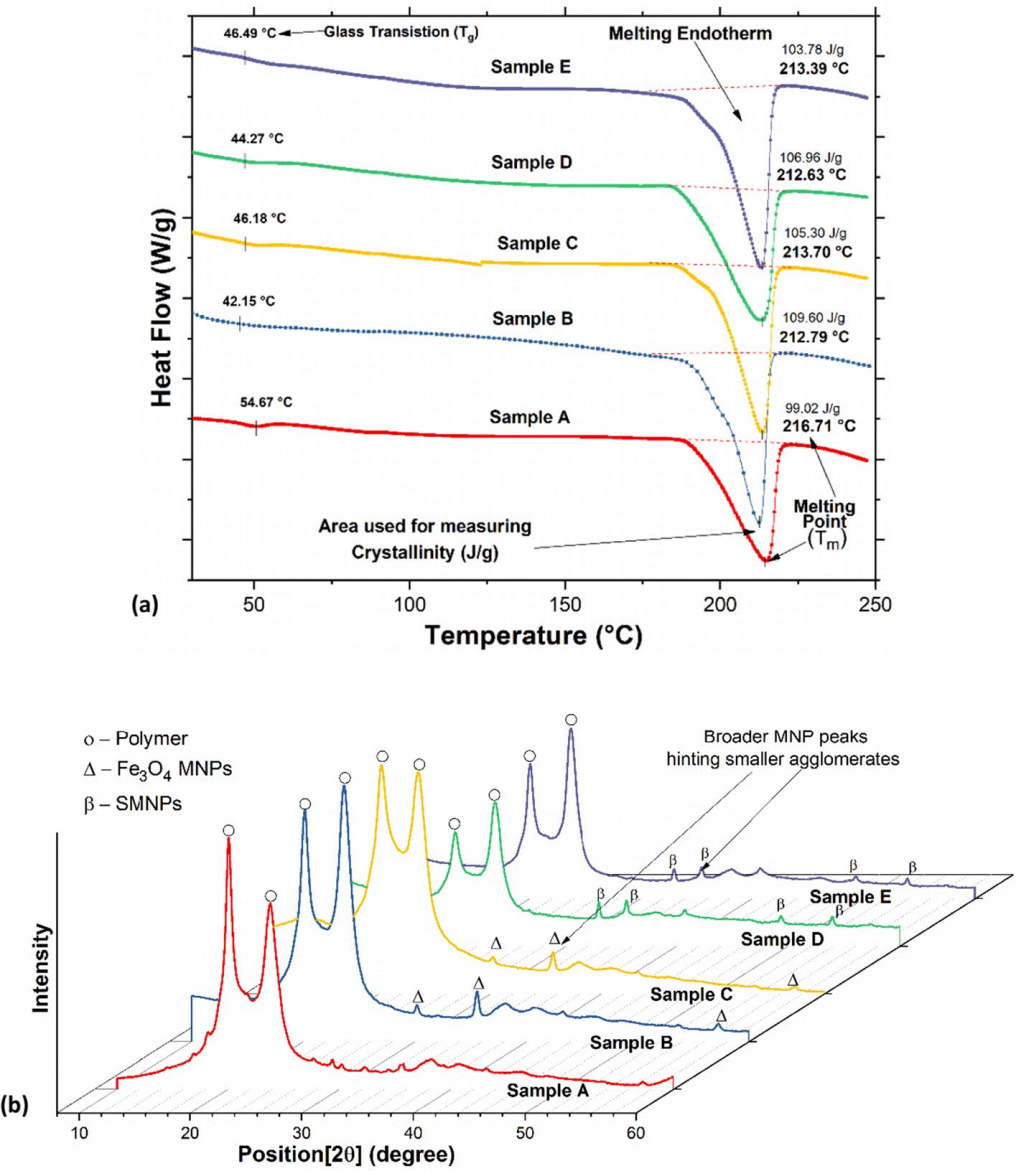
(**a**) DSC curves of all the prepared SHMNP composite samples A to E, showing the glass transition temperatures and melting points and (**b**) XRD patterns of all the prepared SHMNP composite samples A to E.

The differential scanning calorimetry (DSC) results for all the samples, as seen in Fig. 3a, exhibited noticeable endothermic peaks (melting) and clearly depicted the glass transition temperature of all polymer-based samples. The results of the thermal analysis represent the glass transition temperature (T_g_) occurrence of the pristine polymer at ca. 46 °C, which was similar to previously reported values^22^. The melting temperature (T_m_) observed in the DSC results was slightly lower than the published values ca. 220 °C^22^. Additionally, as seen from Table S2 (included in Supplementary Data Section S3), the melting temperature of the SHMNP composite was found to slightly increase for the samples containing silica-coated MNPs. However, the T_g_ values were shown to increase due to the MNP silica coating; the increased wetting of the surface-modified MNPs by the polymer is known to decrease the crystallinity of the nanocomposite and hence increase T_g_ but decrease the melting temperature^23^.

The DSC results also confirmed that wetting of the MNPs by the polymer melt was increased as a result of no particle coating on the MNPs. With no coating on the surface of MNPs, this promoted strong attractive interactions with other particles and in turn with surrounding polymer chains, leading to reductions in cooperative segmental mobility in the polymer and an increase in T_g_^23–25^. The melting endotherm values as presented in the DSC plot in Fig. 3a represent the amount of heat stimuli required to bring about the melt response for each sample. Stating that the samples B to E in real application for structural healing would require minimum heat stimuli of at least 109.60 J/g, 105.30 J/g, 106.96 J/g and 103.78 J/g respectively. Further, information about the crystallinity of nanocomposites was suggested from the XRD results. As seen in Fig. 3b, the main characteristic XRD peaks for the nanocomposite gave a good match to those in the reference pattern JCPDS file number 82-1533; peaks were observed for (hkl) values of (220), (311), (400), (511) and (440) at 2 theta values of 30.3°, 35.4°, 43.1°, 57.3° and 62.7°, respectively. The crystallite sizes of MNPs were estimated from the FWHM of the most intense XRD peaks using the Debye–Scherer formula (as listed in Table S3 in Supplementary Data Section S4).

The two broad crystalline peaks in the XRD data associated with SHMNP materials containing samples C and E showed an increase in crystallinity (sharper peaks) compared to the pristine polymer sample (sample A) and SHMNP samples incorporating uncoated MNPs (samples B and D). This confirmed that the degree of crystallinity changed due to MNPS surface functionalization. Additionally, as observed in Fig. 3b, the silica functionalization of MNPs somewhat suppressed the intense MNP phase-related XRD peaks in samples C and E. In contrast, composites containing samples B and D showed significant broadening and minimal intensity in the comparative XRD data. Once a coating was applied to the MNP, a nonmagnetic silica layer formed on the surface that appears to reduce particle–particle interactions, and hence, the mean crystallite size or agglomerate size was reduced (see Table S3 in Supplementary Data Section S4).

### Magnetic and thermomagnetic response of MNPs and SHMNP composites

**Figure 4:**
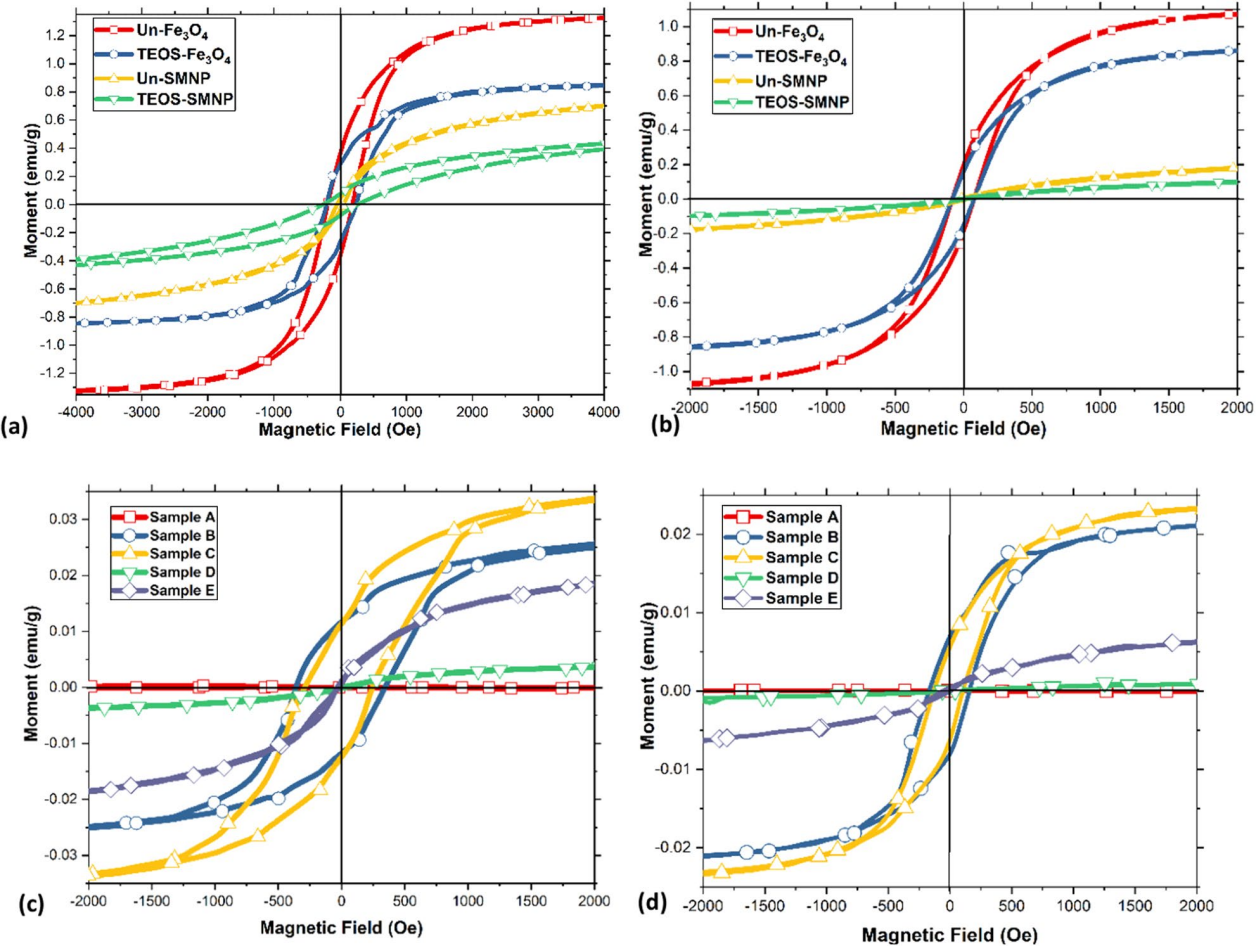
Magnetization hysteresis (M-H) loops for (**a**) MNP samples at 100 K, (**b**) MNP samples at 400 K, (**c**) SHMNP composite samples at 100 K and (**d**) SHMNP composite samples at 400 K.

It should be noted that the particle aggregation effects were particularly apparent only in the case of low MNP contents; hence, the authors only tested the SHMNP composites with 1 wt% MNPs. The effect on the magnetic response suppression due to the silica coating was evaluated and is discussed here. The magnetic properties of the MNPs with and without functionalization and all the synthesized SHMNP composite samples were assessed by magnetization curves at temperatures of 100 and 400 K. The hysteresis loops showed ferromagnetic behaviour, although the variation due to the dispersion of MNPs resulting from silica coating was clearly distinguishable, as seen in Fig. 4a,b. On application of a homogeneous magnetic field of 50,000 Oe (full-scale plots included in Supplementary Data Section S5) at 100 K, the MNPs showed a magnetic moment ratio (M_r_/M_s_) of remanence magnetization (M_r_) to saturation (M_s_), as listed in Table S4 (in Supplementary Data Section S5), of 28.0 and 6.8% for uncoated Fe_3_O_4_ and superparamagnetic MNPs, respectively; for coated Fe_3_O_4_ and SMNPs, it was 34.2 and 7.7%, respectively, at 400 K (as listed in Table S5 in Supplementary Data Section S5), and the ratio was 17.1 and 1.4% for uncoated MNPs and 15.1 and 0.9% for coated MNPs, respectively. Here, the drop in the magnetic moment ratio was observed with the silica coatings of MNPs as a result of suppression of the magnetic remanence caused by the diamagnetic silica layer on the surface.

Furthermore, the SHMNP composite measurements at 100 K (Fig. 4c and Table S6 in Supplementary Data Section S5) showed a magnetic moment ratio of 0% for sample A and 40.9, 31.1, 3.0 and 1.5% for MNP samples B, C, D and E, respectively. The measurements at 400 K (Fig. 4d and Table S7 in Supplementary Data Section S5) revealed magnetic moment ratios of 33.5, 25.1, 19.6 and 2.8% for MNP samples B, C, D and E, respectively.

After increasing the temperature to 400 K, the M_r_/M_s_ ratio decreased significantly for all samples compared to small reduction observed for sample D (Fig. 4c,d), suggesting an uneven dispersion or agglomeration of MNPs. The high degree of agglomeration in sample D was further confirmed by the XRD data and small-angle scattering results. The observation of the wide hysteresis loop at the lower temperature of 100 K (compared to that at the higher temperature of 400 K) hinted towards the superparamagnetic behaviour induced in the designed SHMNP composites^19^. The SMNP samples (D and E) showed almost zero coercivity and a significant superparamagnetic response^26^ compared to samples B and C, which contained Fe_3_O_4_ nanoparticles. The hysteresis loops of the Fe_3_O_4_ SHMNP composites revealed symmetric behaviour similar to that of ferromagnetic materials^27^, confirming the suitability of the synthesized SHMNP composites for magnetic stimuli-based self-healing applications in structural composites. The magnetic remanence in the silica-coated SMNP-containing sample E was almost zero (ca. 2–3%) compared to the sample containing the coated Fe_3_O_4_ (sample C). This suggested that the former SHMNP composites (sample E) had a short Neel relaxation time in response to the applied magnetic field. The coercivity of all Fe_3_O_4_ SHMNP composite samples (samples B and C) had values in the ranges of 270–310 Oe and 110–120 Oe (except the pristine sample, which showed zero magnetization) at temperatures of 100 and 400 K, respectively. However, the coercivity for all SHMNP composite samples (samples D and E) was low because of the decrease in the strength of the dipole–dipole interaction.

**Figure 5:**
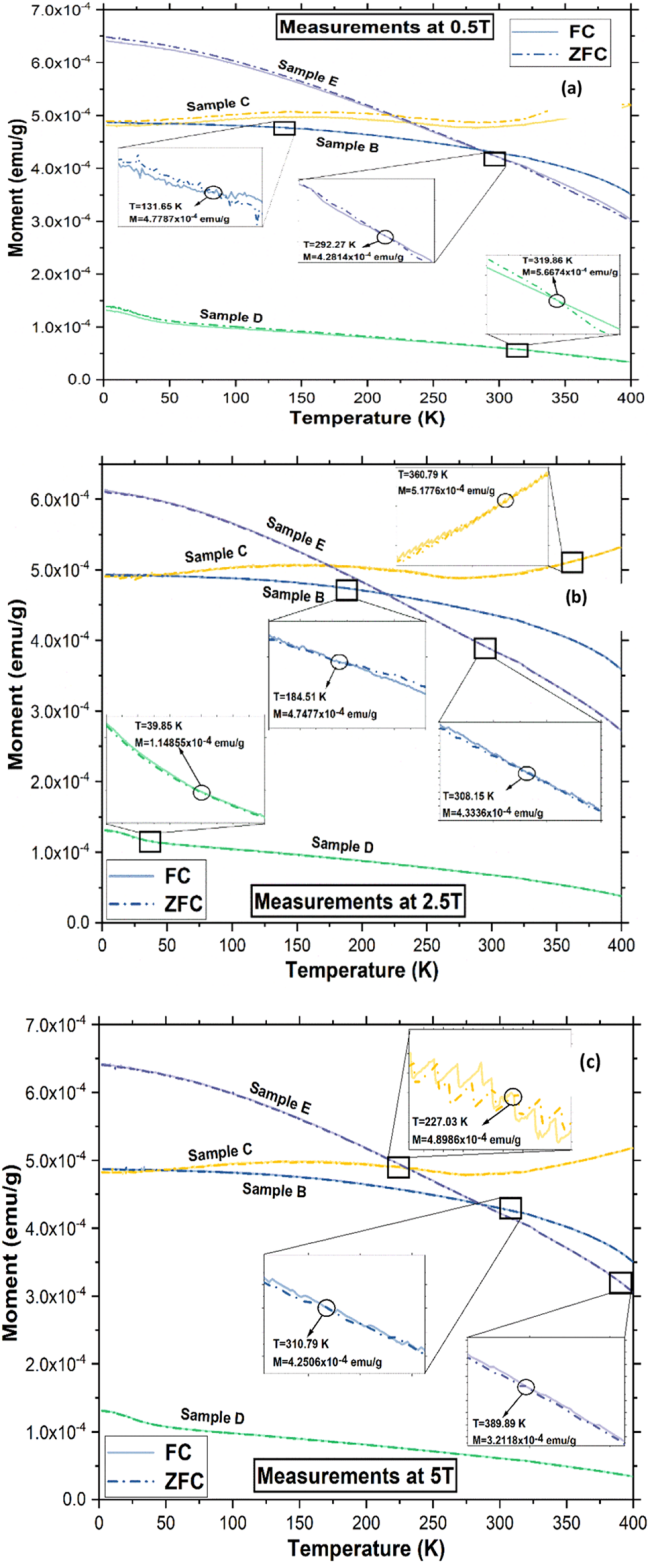
Field cooled (FC) and zero field cooled (ZFC) response for SHMNP composite samples B, C, D and E at (**a**) 0.5 T, (**b**) 2.5 T, and (**c**) 5 T.

The FC/ZFC (field cooling/zero field cooling) curves for all the SHMNP composite samples at 0.5, 2.5 and 5 T magnetic fields are shown in Fig. 5. Variation in the magnetic field showed a small divergence between the ZFC and FC curves (zoomed in inset figure), which occurred in the temperature range of 132 to 320 K. As the field strength was increased, the ZFC–FC crossing temperature increased for SHMNP composite samples B, C and E; however, the trend was absent in SHMNP composite sample D due to high agglomeration. The Curie temperature can be determined from d^2^M/dT^2^ calculations. These values are included in Fig. 5; however, small peaks other than Curie temperature peaks were also observed, which can be attributed to the polymer matrix surrounding the MNPs. Typically, FC/ZFC curves provide information about effective anisotropy (K_eff_), which encompasses crystal, shape, and surface anisotropy in a single parameter. However, there was a remarkable absence of the Verwey transition, a metal-to-insulation transition of MNPs at a certain temperature range, often associated with a downward or upward change in the coercive field (Hc) values^28^, which was found to be absent for SHMNP composite samples D and E. This implied that the reduction or complete loss of the Verwey transition for SMNPs was possible if they were added into a polymer matrix. For instance, some recent reports indicate a strong reduction^26^ or even a complete loss^27^ of the Verwey transition temperature in SHMNP composites. Furthermore, there were signs of disappearance of hysteresis in Fig. 4c,d for SHMNP composite samples B and C and SHMNP composite sample D, indicating that they had almost become magnetically unblocked, almost reaching a thermal equilibrium state at lower temperatures. In contrast, SHMNP composite sample E showed a distinct behaviour compared to the other SHMNP composite samples (its FC and ZFC curves gradually approached each other), and the observed blocking temperature was higher at temperatures near 400 K, where both curves crossed each other. The increase in field strength was directly proportional to the blocking temperature for SHMNP composite samples B and E; however, this was not true for other SHMNP composite samples B and E, likely due to the inhomogeneous dispersion of MNPs in the weakly paramagnetic polymer matrix. The trend shows that the blocking temperature increased at a higher field strength, which was helpful in melting the polymer surrounding the dispersed MNPs without their transition to the paramagnetic phase, therefore stopping heat production. This is useful for self-healing SHMNP composite applications where localized polymer melting is the primary aim^3^. These data can be used for calculating the particle distribution based on the magnetic response of the SHMNP composite sample and were added to support the SHMNP composite model using simulated data. To understand the effect of material type and dispersion state on the magnetocaloric properties of the SHMNP composite, an interaction radius model was constructed.

### Interrelation of the dispersion state of MNPs with the magnetic response

The equivalent diameter of the ellipsoidal nanoparticles within the SHMNP composites was estimated using ImageJ software and following the method explained elsewhere^29,30^. The estimated average diameter (Table S8 in Supplementary Data Section S6) for each sample was then used as input data in MATLAB^®^ code to simulate the 3D model.

**Figure 6:**
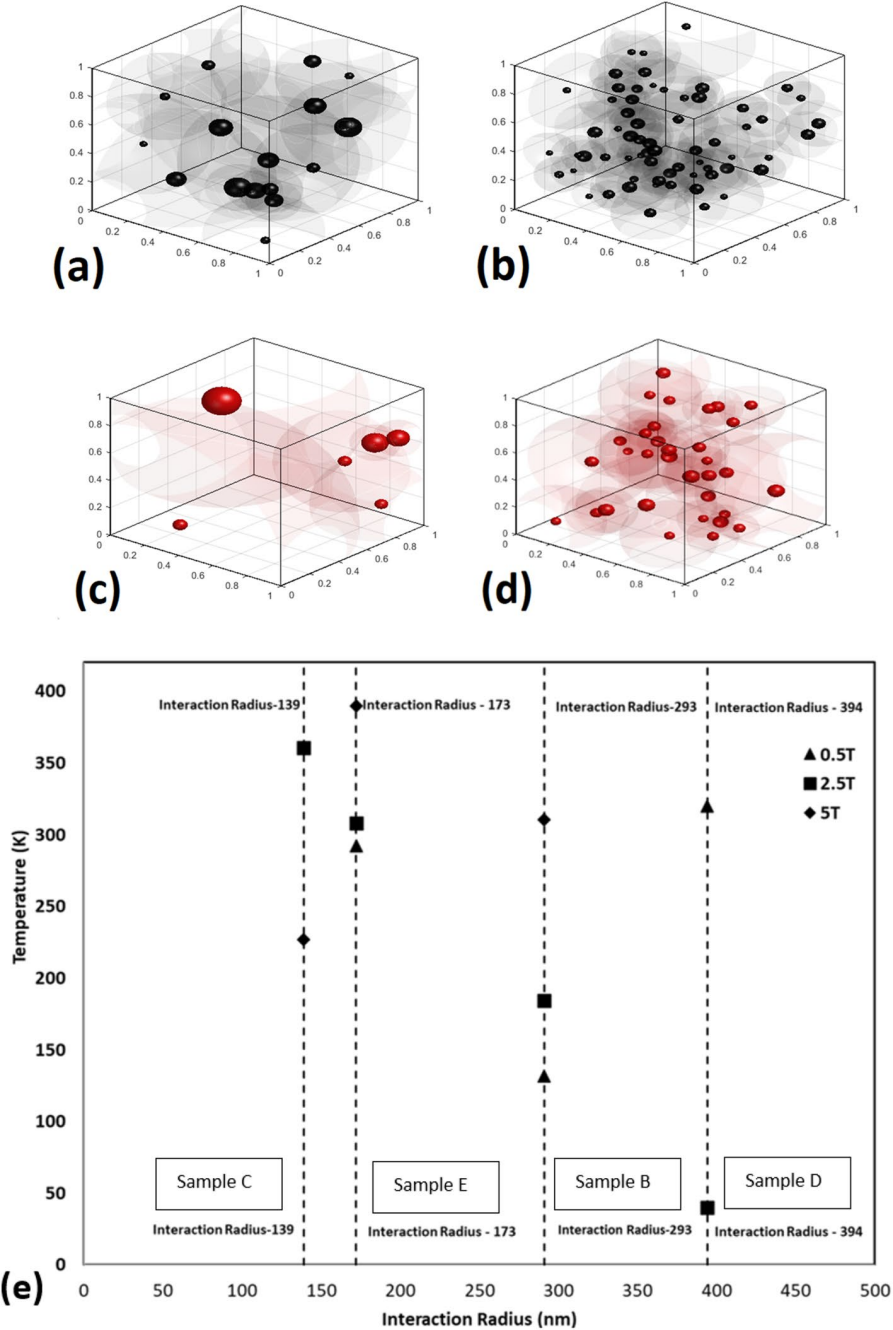
Simulated representation included with the interaction radius (IR) of the individual nanoparticle/agglomerates present in the synthesized nanocomposite (1 μm^3^) for Fe_3_O_4_ samples (**a**) sample B, (**b**) sample C and superparamagnetic samples (**c**) sample D, (**d**) sample E, respectively (herein, black spheres represent Fe_3_O_4_ and red spheres are for superparamagnetic nanoparticle/agglomerates, respectively.) (**e**) Correlation between blocking temperature and interaction radius of the SHMNP samples.

To simplify the simulated model, all the entities were generated as only spherical nanoparticles/agglomerates. The simulated models for all the SHMNP composite samples are included in Supplementary Data Section S6, where each sphere represents the small- to large-size agglomerates of MNPs. The purpose of this modeling was to visualize and interpret the dispersion state of the synthesized SHMNP composite samples with and without the MNP surface functionalization effect, which was quite challenging to obtain with physical characterization methods alone. The magnetic interaction (either dipolar or exchange) between neighbouring MNP agglomerates in each SHMNP composite sample was estimated by calculating the interaction radius (IR) for each nanoparticle/agglomerate generated as individual spheres of variable sizes in the model, as seen in Fig. 6a–d. The IR values, summarised in Table 2, considered the nearest neighbor condition, i.e., the distance between the nearest neighbouring agglomerates in the model. These data were used to graphically represent the interaction region, as shown in Fig. 6a–d, and subsequently, they were correlated with the magnetic behavior of all SHMNP composite samples in Fig. 6e.

**Table 2:** Summary of the calculated interaction radius values for each sample.

Sample	Minimum interaction radius (nm)	Maximum interaction radius (nm)	Average interaction radius (nm)	Standard deviation (nm)
Sample B	123	448	291	109
Sample C	77	345	139	55
Sample D	117	806	394	262
Sample E	65	371	172	78

Interestingly, the simulated models demonstrate that the IR was a variable term dependent on the size of the agglomerates and on the nearest possible neighbours. From Table 2, it can be observed that the minimum and maximum IR values for all SHMNP composite samples were reduced by ca. 50 and 430 nm, respectively, as a result of MNP coating.

Interrelating the IR data of all the samples with that of the observations from TEM micrographs, SAXS/WAXS and XRD data, the results combined suggest that functionalization (silica coating) of the MNPs was more effective in reducing agglomeration within the polymer matrix in the case of Fe_3_O_4_ than that of the SMNPs. The possible reason for this can also be that the dipole–dipole type interparticle attraction among the silica-coated SMNPs was higher than that among the coated Fe_3_O_4_ MNPs.

A high blocking temperature is required for applications such as structural self-healing, where the surrounding polymer matrix should melt to heal cracks and deformities in the material structure. If heating of the MNPs stops at lower temperatures, the materials will not melt high modulus polymers such as PA6. The requirement of achieving high temperature overrides the efficiency goal. To assess the key parameters required to achieve the requirement, the data on the interaction ratio (IR) and blocking temperature values were used.

As seen in Fig. 6e, the value of blocking temperature derived from the ZFC–FC magnetisation data is plotted against the IR for various applied magnetic fields. Low values of IR seemed to be related to higher blocking temperatures. Even with the higher IR value of 173, the blocking temperature for SHMNP composite Sample E was found to be mostly higher than the temperature for SHMNP composite sample C with IR -139 at all applied magnetic field strengths except 2.5 T. The IR value difference between SHMNP composite samples C and E was small enough; however, SHMNP composite sample D (containing uncoated SMNP) with a high IR value of 394 showed a low blocking temperature compared to SHMNP composite sample B (containing uncoated Fe_3_O_4_ MNPs) under an applied field strength of 2.5 T. This could be attributed to an inhomogeneous external magnetic field. From these observations, it can be deduced that the low coercivity and superparamagnetic behaviour of SMNPs in SHMNP composite samples E and D contributed to higher blocking temperatures. When considering the magnetic properties of the MNPs (superparamagnetic, low coercivity), the IR value was a good parameter to determine the efficiency of magnetocaloric properties. Looking at the IR values in Table 2, it seems that particle dispersion also played an important role, along with the intrinsic properties of the filler material. The blocking temperature values seem to be related to the coercivity values rather than the saturation magnetization. SHMNP composite sample E displayed low IR values and showed a roughly linear increasing trend compared to SHMNP composite samples D and B (with uncoated MNPs), which was associated with the greater particle agglomeration in SHMNP composite samples D and B. Moreover, magnetocaloric effects are known to be related to coercivity values rather than the magnetic saturation value for most materials^31^. These low coercivity MNPs and their dispersion state^32^ in the synthesized SHMNP composites determined the resulting magnetocaloric behaviour. However, it was not applicable to MNPs with a size greater than 100 nm^33^. For nanocomposites, an efficient design requires good dispersion and low coercivity materials.

The amount of heat generated is correlated to the change in the magnetic entropy in the system^34^. To estimate the heat generation, the change in entropy ΔSm was approximated using the isotherm M-H curves^35^ at 100 K and 400 K. Using the limited data, the calculated values of ΔSm were found to be highest for SHMNP and SHMNP composite with magnitude in region of 20.5 J K^−1^ kg^−1^ and 0.1 J K^−1^ kg^−1^, respectively. The values for change in entropy demonstrates that SHMNP and SHMNP composite, both will generate highest heat in response to alternating magnetic field.

### Correlation between magnetic properties and thermomagnetic properties

As seen in the Fig. 5a–c, the magnetic moment for sample E (superparamagnetic materials) was found to be highest at 2.5 T amongst all samples. Moreover, the low coercivity and small area enclosed by the M-H hysteresis curve shows low losses for the sample E, which has a highest magnetisation 6.5 × 10^–4^ emu/g at lowest temperature. The trend showed that the lower the coercivity or remanence of nanoparticles, higher the magnetisation in relation to temperature. The excellent magnetocaloric effect depends on the heat capacity Cp (T) and isothermal change in the magnetic contribution into the entropy ΔSm^36^. The ΔSm is proportional to the difference between the measured magnetisation values at lowest and highest temperature. From the Fig. 5, it can be concluded that the low coercivity of the nanoparticle materials is responsible for generating the higher ΔSm in the material and thus, the higher temperature generation in response to exposure of the alternative magnetic field^36^.

## Conclusions

Using a viable method, the correlation between the magnetic properties of SHMNP composites, the dispersion state and filler material of MNPs, was established, and a clear understanding of the interaction between the dispersion state and the material properties in the SHMNP composites was developed. 3D modeling of the MNP dispersion and magnetic properties of the SHMNP composites showed that a lower calculated interaction ratio equated to a better dispersion. The TEM, XRD and SAXS characterization results showed that silica functionalization on the MNPs reduced the interaction ratio and, therefore, improved the dispersion of the filler materials. To prepare self-healable PA6 nanocomposites suitable for structural applications, it is essential to coat the MNPs such that they provide good dispersion but retain their magnetic properties. Moreover, to achieve high temperature in composites for local melting of the polymer phase (for self-healing of the structure), low coercivity MNPs should be used. The SHMNP composite prepared using the silica-coated SMNPs (containing particles with superparamagnetic behaviour) showed exceptional magnetic behaviour compared to any other magnetite-containing SHMNP composite samples reported, with almost zero coercivity and the least remanence magnetization (including enhanced superparamagnetic response) compared to the paramagnetic Fe_3_O_4_ SHMNP composites. Such a material with a low interaction radius is presumed to be the most suitable for self-healable polymer nanocomposites suitable for structural applications.

## Supplementary Information

Supplementary Information.

## Data Availability

All data needed to evaluate the conclusions in the paper are presented in the paper and/or the Supplementary Materials. The datasets generated and/or analysed during the current study are available in the public OpenAir repository, https://rgu-repository.worktribe.com/output/1579700.
