# Supplementary material for: Tuneable magnetic nanocomposites for remote self-healing
Source: Sci Rep. 2022 Jun 17;12:10180. doi: 10.1038/s41598-022-14135-8 (PMC9205898; doi:10.1038/s41598-022-14135-8)
Supplement: Supplementary file 1 — Supplementary Information. [file 41598_2022_14135_MOESM1_ESM.docx]

**Supplementary Data**

**Tuneable Magnetic Nanocomposites for Remote Self-Healing**

**Ranjeetkumar Gupta^1^, Priya Gupta^2^, Charles Footer^3^, Gavin B. G. Stenning^4^, Jawwad A. Darr^3^* and Ketan Pancholi^1^***

^1^ Advanced Materials Group, School of Engineering, Robert Gordon University, Aberdeen AB10 7GE, UK

^2^ Lakshmi Narain College of Technology & Sciences, RGPV, Indore, MP, India.

^3^ University College London, Department of Chemistry, 20 Gordon Street, WC1H OAJ, UK.

^4^ ISIS Neutron and Muon Facility, STFC Rutherford Appleton Laboratory, Didcot OX11 0QX, UK

^*^Correspondence: k.pancholi2@rgu.ac.uk Tel.: +44-1224-262317; j.a.darr@ucl.ac.uk Tel: 020 7679 4345

# **Section 1 (S1):** Materials and Methods

The steps involved in preparing the polymer magnetic nanocomposite (PMC) samples and their characterisation/testing methods are mentioned in this section.

1.1 Materials

Iron oxide MNPs (<50 nm particle size), Citric acid 99%, ε-caprolactam (CL) (99% purity), 3.0 M Ethyl Magnesium Bromide (EtMgBr) solution in diethyl ether, N-Acetyl Caprolactam (NACL) (99% purity), Ammonia solution 25% and Tetraethyl orthosilicate ≥99.0% GC (TEOS) were purchased from Sigma-Aldrich Company Ltd. Dorset, UK and used as received. Proprietary superparamagnetic nanoparticles were used as received from UCL. These are known as “SMNP” in the manuscript and were made using a process similar to that described in the experimental methods section below. The particles were characterised by having a particle size of 30 nm rhombic crystals. Deionized water with 18 MO conductivity was used throughout the experiment.

1.2 Experimental methods

1. *Synthesis of SMNP nanoparticles*

A schematic diagram of the reactor setup is shown in Figure S1. Two pumps (Primeroyal K, Milton Roy, Pont Saint-Pierre, France) were used to provide the supercritical water (containing 0.5 M H_2_O_2_) and base (1.0 M KOH) feeds at 80 and 40 mL min^-1^, respectively (pumps P1 and P3). 10 MΩ/cm deionized water purified using a Millipore Elix® Essential water purification system was fed from pump P1 and heated to 450 °C in flow using a 7 kW custom-built electrical water heater. The metal precursor feed was pumped by P2, delivering a total flow rate 40 mL min^-1^. The premixed precursor solutions consisted of the desired stoichiometries of each metal nitrate precursor, with a total metal salt concentration of 0.15 M. The metal precursor feed delivered from pumps P2 was first mixed with the 1.0 M KOH base feed in flow (from pump P3), before the combined mixture was introduced to a stream of supercritical water (from pump P1) in a patented Confined Jet Mixer (CJM)^1, 2^. The reaction of the precursor solutions in the CJM, resulted in the rapid crystallization of nanoparticles. The particle-containing aqueous flow was then cooled to ca. 40 °C using a 1.5 m pipe-in-pipe heat exchange column, before passing through a back-pressure regulator (BPR). The resultant nanoparticle slurries were collected in beakers and were then cleaned by repeated centrifugation and washing with deionized water until the supernatant had conductivity below 50 µS/cm as measured using a conductivity probe (model HI98311, Hanna Instruments, Leighton Buzzard, UK). The concentrated, cleaned slurry was then freeze-dried by slowly heating from -60 °C to 25 °C, under a vacuum of <13 Pa, over 24 h using a Virtis Genesis 35XL freeze drier.


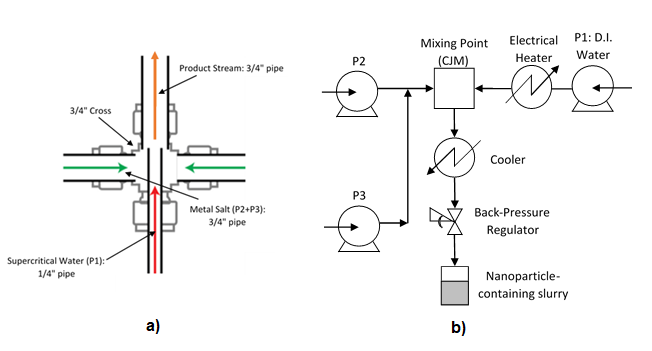


Figure S1. a) Diagram representing the confined jet mixer CJM. b) Schematic of the CHFS process, which demonstrates how the heated water from P1 is combined with the aqueous precursors from P2 and P3 at the CJM mixing point.

1. *Optimised silica functionalisation of the MNPs.*

The as-received MNPs (both SMNP and Fe_3_O_4_) weighing 2.0 g were added to 65 mL of aqueous citric acid (0.5 g/mL concentration), associated with rigorous stirring. For effective adsorption, the pH value was adjusted to 5.2 by adding aqueous ammonia solution, which resulted in dissociation of two carboxylic groups of each of the citric acid molecules. Further adsorption was enhanced by heating to 80 °C and rigorous stirring continued for 90 min. After that, the pH was increased to 10.1, wherein the third carboxylic group of the adsorbed citric acid, was dissociated^3^. The resulting nanoparticles’ have higher surface charge aiding electrostatic inter-particle repulsion and preventing agglomeration. Excess solvent was removed by centrifuging at 5000 rpm for 5 min and the collected nanoparticles were dispersed in clean DI water.

The Stöber functionalisation method was used to nucleate a silica layer on both types of MNP surfaces by first hydrolysis and then polycondensation based deposition of TEOS. It’s a well-known that the hydrolysis reaction can be catalysed using acid or alkaline solvent media, whereas it is very slow in neutral conditions^4^; so aqueous NH_3_ as an alkaline catalyst was used herein. The amount of TEOS to be added was calculated considering 2.4 mass ratio of MNP:TEOS. 0.85 g of TEOS dissolved in ethanol was added to the prepared suspension of magnetite MNPs; considering that the average diameter of coated MNPs are 30 nm and the used mass ratio resulted in a functionalised layer of *ca*. 2 nm (based on the surface area estimation). The pH was then stabilised at 12.0 by adding aqueous NH_3_ which triggered the Stöber functionalisation reaction. The mixture was rigorously stirred for 3 hr at room temperature (RT) with probe-type 150-W sonicator (Soniprep 150; MSE., UK), giving the MNPs a rough silica-functionalised coating. The MNPs were centrifuged at 5000 rpm for 5 min and then washed thoroughly three times with DI water. Collected MNPs were dried in oven at 120 °C under vacuum and further sieved. The finely sieved MNPs were then used in the in-situ polymerisation of the magnetic PMC synthesis. The optimised parameters of the activator and initiator used in the in-situ polymerisation were followed from a previous report^5^. The summary of the processing technique is presented in Figure S2.


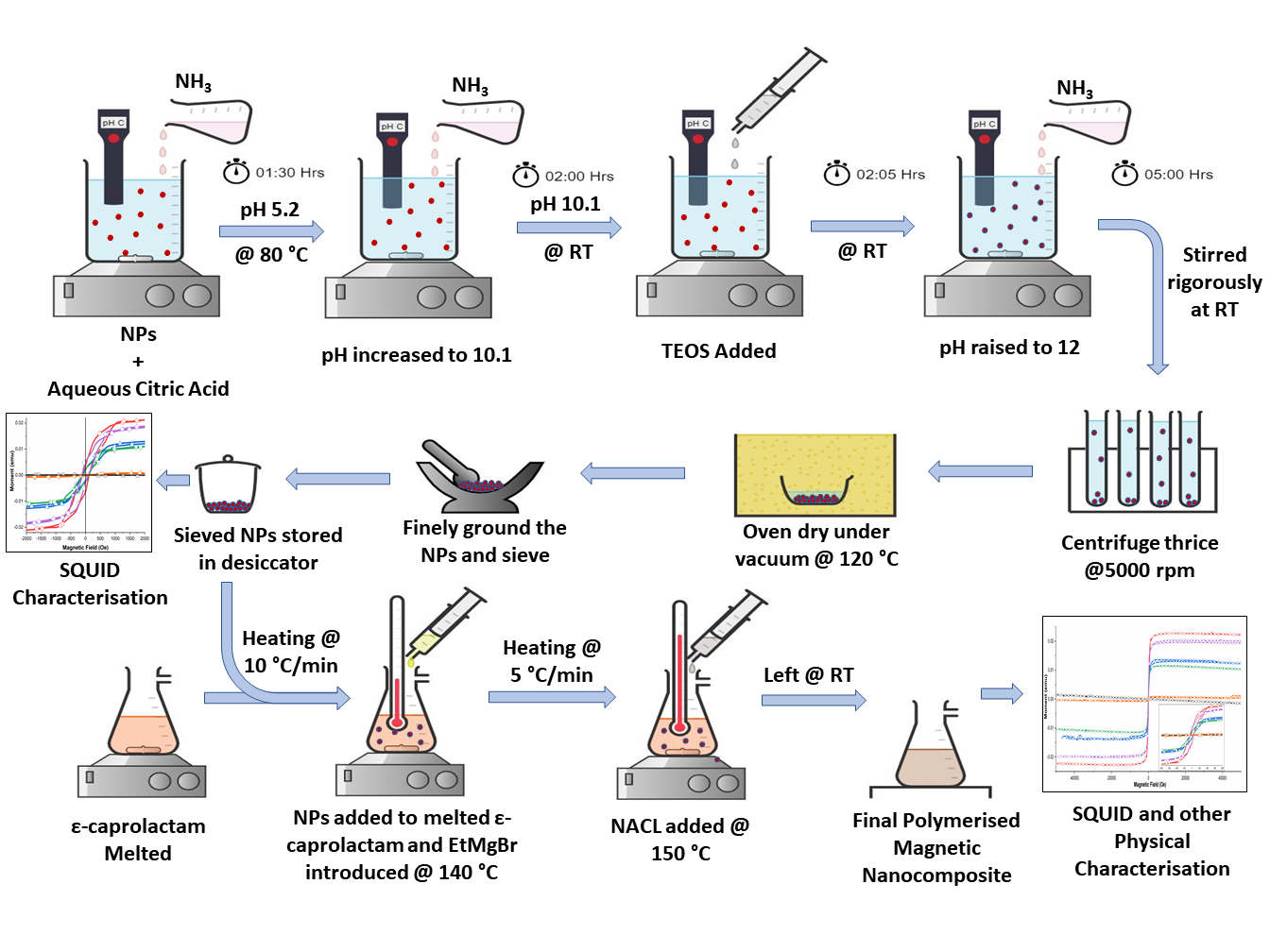


**Figure S2.** Experimental scheme that was followed for the functionalisation of the both types of MNPs and its subsequent usage in the in-situ polymerisation in the manufacture of magnetic nanocomposites.

1. *Characterisation methods for the functionalised MNPs and synthesised PMCs*

Total four samples, as summarised in Table 1, were prepared for the characterisation study. Two samples containing 1 w/w % of silica coated SMNPs and two samples containing silica coated Fe_3_O_4_ were characterised using ATR-FTIR (Attenuated Total Reflection- Fourier Transmission Infrared Spectroscopy) to determine its chemical composition and confirm the successful polymerization of CL forming PA6 polymer. Previous studies have shown that the dispersion state of MNPs in the polymer, modifies the agglomerate size of the particles and degree of crystallinity of the resulting nanocomposite. To determine effect on degree of crystallinity and crystallite size, all samples were characterized using Differential Scanning Calorimetry (DSC), SAXS/WAXS and subsequent X-ray Diffraction (XRD).

1. FTIR-ATR

The nanocomposite samples were characterized using Perkin-Elmer ATR-FTIR (Attenuated Total Reflection- Fourier Transmission Infrared Spectroscope) Spectrum Gx system containing DGS-KBr sensor to identify phases and structural changes after addition of the iron oxide MNPs. In order to scan each sample, the nanocomposite films of approximately 0.1 mm thickness were prepared and total 30 scans in range of 525 to 4000 cm^-1^ were carried out at a resolution of 4 cm^-1^. The gain was set to 2, whereas the optical velocity was fixed to 0.4747 m/s.

1. DSC

DSC was performed using a TA Instruments DSC Q100 at a heating rate of 10° C/min under a nitrogen environment with a temperature range of 20 to 270°C using a sample mass of 9 mg. The Heat/Cool/Heat standard cycle type analysis was selected for accurately depicting the behaviour and T_g_ and T_m_ for the samples prepared. The running segment consisted of a ramp heating at 10 °C/min to 250 °C, then ramp cooling at 5 °C/min to -90 °C and finally ramp heating at 10 °C/min to 250 °C. The glass transition temperature (T_g_) and melting temperature (T_m_) were determined from the DSC traces obtained, where the first small endothermic peak represents the glass transition temperature, and the second larger endothermic peak represents the melting temperature of the nanocomposite sample.

1. XRD

A PANalytical X'Pert Pro MPD, powered by a Philips PW3040/60 X-ray generator and fitted with an X'Celerator detector was used. Diffraction data is acquired by exposing samples to Cu-Kα X-ray radiation, which has a characteristic wavelength (λ) of 1.5418 Å. X-rays were generated from a Cu anode supplied with 40 kV and a current of 40 mA. The data were collected over a 2θ range of 0 to 80° with a step size of 0.117° (2θ) and nominal time per step of 1099.82, using the scanning X’Celerator detector. Fixed anti-scatter and divergence slits of 0.38 mm were used together with a beam mask of 10 mm and all scans were carried out in a continuous’ mode. Phase identification was carried out by means of the X'Pert-PRO accompanying software program PANalytical High Score Plus in conjunction with the JCPDS card.

1. TEM

TEM images were used to determine the morphology and mean diameter in the MNP agglomerates. TEM imaging was conducted at an accelerating voltage of 100 kV, with a spot size of 10 nm. The exposure time was varied from 0 to 50 s. The images of all samples were obtained using a Philips CM100 TEM at different direct magnifications, ranging from x7900 to x245000. An ultrathin section of nanocomposite obtained using microtome were placed on gilder grid of 400 mesh to obtain all images. For MNP imaging, the particles were dispersed in isopropyl alcohol and droplet were placed on TEM grid.

1. SAXS/WAXS

SAXS and WAXS scattering patterns were obtained on Xenocs Nano-inXider, equipped with microfocus sealed tube: Cu, 30W point focus. With Dectris Pilatus 3 hybrid photon counting (two fixed) detectors for continuous and simultaneous SAXS and WAXS acquisition up to 2θ = 60°. The beam path was windowless beam path, entirely under vacuum from beam delivery system to detector sensor. The SAXS patterns were obtained over a scattering vector length within the range of 0.008 Å^-1^ < q < 0.18 Å^-1^ and WAXS patterns with the range of 0.18 Å^-1^ < q < 0.24 Å^-1^. One-dimensional (1D) fitting of the scattering curves was obtained by an azimuthal binning and averaging of corresponding two-dimensional scattering patterns using the XSACT (X-Ray Scattering Analysis and Calculation Tool) supplied with the instruments.

1. Magnetic characterisation

Magnetisation loops of the synthesised composite samples were measured at T = 100 K and 400 K on MPMS3 squid-VSM, integrated with Superconducting Quantum Interference Device (SQUID) detection system and precision temperature control unit. The instrument had temperature range of 1.8 to 400 K and applied maximum field strength of ±7 Tesla with filed uniformity of 0.01% over 4 cm.

1.3 Building the 3D model interrelating with the size distribution data from physical characterisations

The TEM, XRD and SAXS results comparatively gave an estimation of the MNP/agglomerate particle size in the dispersion state. This data was input to the simulated 3D model build using a MATLAB^®^ platform. The TEM micrographs firstly were processed in Photoshop^®^ software; wherein they were cropped, rotated, and enlarged, for removing their edges and any distorted background. Further, they were digitally enhanced by filtering techniques for several purposes, such as removing the background noise and their artifacts or improving upon the definition and sharpness of the image. The imaged were equalized, correcting the brightness and contrast of the grayscale images, ensuring proper black and white tonalities were reached to distinguish the polymer matrix (represented by the grayscale region) and the pure black entities (representing the magnetic nanoparticles). Finally, the processed images were manually coloured, with the polymer background toned with yellow and green shade and the black nanoparticle entities toned with black and red colour representing Fe_3_O_4_ and CHFS made SMNP MNPs/agglomerate, respectively. Then the final toned images were saved as high quality 8-bit TIFF format and loaded in ImageJ image processing software for estimating the nanoparticles/agglomerates sizes of all the sample variations. This size data was then fed to the custom designed MATLAB^®^ code, to be used as a basis for generating the random nanoparticles/agglomerates sizes for the simulated 3D model of the nanocomposite. The code generates the model with the required wt% of nano-inclusions and MNP/agglomerate sizes within the fed diameter range. Similar image tone allocation was followed in the processed TEM micrographs (in the simulated model as well), with black and red colour representing Fe_3_O_4_ and CHFS SMNP nanoparticles/agglomerates, respectively.

**Section 2 (S2):** **Detailed analysis of SAXS data.**

All obtained spectra were corrected for background scattering before any further analysis. The relation is represented by Equation S1^6^ as follows:

$I\left( q \right)=G\exp\left( -\frac{q^{2}R_{g}^{2}}{3} \right)$ Eqn. S1

herein, G is the Guinier pre-factor and R_g_ the radius of gyration. The Guinier plot represented in Figure S3 of Section 2, of Ln I(q) vs q^2^, is used to calculate the slope of the chosen region that dictates the value of R_g_, giving out the MNP/agglomerate size qualitatively.





(a)





(b)

**Figure S3**. Guinier plot for all the samples (a), with the region fitting highlighted (b).

The MNPs are assumed as perfect sphere and the diameter D is calculated with the Equation S2^6^:

$D=2\times\left( 5/3 \right)^{1/2}R_{g}$ Eqn. S2

The slope of regions in the Guinier plot were calculated to give the R_g_ estimate^7^, this was used to calculate the diameters of the MNP/agglomerate using Equation S2. The calculated values of diameters of the MNP/agglomerate for all the SHMNP samples is summarised in Table S1 below.

**Table S1:** Summary of the calculated average sizes of the MNPs in each SHMNP composite sample

|  |  | sample B | sample C | sample D | sample E |
| --- | --- | --- | --- | --- | --- |
| 1^st^ Region Fitting | I(q) Range | 0.009-0.012 | 0.009-0.014 | 0.009-0.013 | 0.009-0.012 |
|  | R_g_ | 198 ± 9 | 150 ± 6 | 199 ± 10 | 154 ± 8 |
|  | D(nm) | 58 ± 9 | 37 ± 6 | 60 ± 10 | 40 ± 8 |
| 2^nd^ Region Fitting | I(q) Range | 0.012-0.018 | 0.014-0.021 | 0.013-0.018 | 0.012-0.021 |
|  | R_g_ | 143 ± 6 | 94 ± 8 | 145 ± 5 | 116 ± 5 |
|  | D(nm) | 36 ± 6 | 25 ± 58 | 37 ± 5 | 29 ± 5 |
| 3^rd^ Region Fitting | I(q) Range | 0.018-0.022 | - | 0.018-0.022 | - |
|  | R_g_ | 97 ± 4 | - | 90 ± 3 |  |
|  | D(nm) | 25 ± 4 | - | 24 ± 3 |  |

**Section 3 (S3): Degree of crystallinity analysis for all samples using DSC data.**

The enthalpy of all the samples were calculated using the Universal Analysis software that comes along with the DSC instrument control package. By quantifying the heat associated with the melting endotherm. This heat was then reported in terms of percent crystallinity by normalizing the observed heat of fusion with that of the 100% crystalline PA6 polymer. The area used for the enthalpy (crystallinity) calculation as identified using the “Integrate Peak” functionality of the TA Universal Analysis 2000 software^8^ is recreated in the plots in Figure 3 (a) (included in the main text). The same analysis also helped identify the “Melt Peak Temperature” of the endotherm peak, which was the melting point T_m_ of the samples and listed in the Table below. Adding to the discussion, the Glass transition temperature T_g_ was also identified using the “Glass/Step transition” functionality available in the same software.

The degree of crystallinity for all the samples was calculated from the following Equation 3^8^, using the standard reference value of PA6 as cited in the main text.

$Sample Degree of Crystallinity= \frac{Sample Enthalpy from DSC plot}{PA6 Enthalpy from Reference Text} \times100\%$ Eqn. S3

**Table S2**: A list of degree of crystallinity, Glass transition (T_g_) and Melting temperature (T_m_) from DSC results.

| Sample | T_g_ (°C) | T_m_ (°C) | Enthalpy (J/g) | Degree of Crystallinity (%) |
| --- | --- | --- | --- | --- |
| *Sample A* | 54.7 ± 2 | 216.7 ± 3 | 99.0 ± 2 | 52.1 ± 2 |
| *Sample B* | 44.3 ± 2 | 212.8 ± 3 | 97.6 ± 4 | 51.4 ± 4 |
| *Sample C* | 46.2 ± 2 | 213.7 ± 2 | 95.3 ± 6 | 50.1 ± 6 |
| *Sample D* | 44.3 ± 1 | 212.6 ± 2 | 96.9 ± 3 | 51.0 ± 3 |
| *Sample E* | 46.5 ± 1 | 213.4 ± 3 | 93.8 ± 2 | 49.3 ± 2 |

**Section 4 (S4): Crystallite size calculation for all samples using XRD data.**

The crystallite sizes of MNPs were calculated from FWHM of the most intense peaks using the Scherrer formula as shown in Equation S4^9^. The Scherrer equation gives the relation between the peak width(B) and the crystallite size(L). It states that peak width is inversely proportional to crystallite size.

$B\left( 2\theta\right)=\frac{K\lambda}{L\cos\theta}$ Eqn. S4

**Table S3**: Crystallite sizes of MNPs calculated from FWHM of intense peaks observed in XRD.

| Sample Type | Absolute Crystallite Size | |
| --- | --- | --- |
|  | Size(A) | Size(nm) |
| *Sample B* | 456.9 | 45.7 |
| *Sample C* | 340.2 | 34.0 |
| *Sample D* | 514.7 | 51.5 |
| *Sample E* | 368.8 | 36.9 |

Ideally, PA6 contains two dominant monoclinic crystalline phases that are usually referred to as the α-phase and γ-phase. In the α-phase, which is known to be the most stable phase in terms of thermodynamics, the hydrogen bonds appear in between adjacent antiparallel chains, with the entirety of the phase attaining a trans-chain conformity^10^. This, however, is not the case in the γ-phase, as chains are seen to appear twisted in order to enable the formation of hydrogen bonds between parallel chains. Two broad peaks were seen to appear around 4.2 A° (21° 2θ) and 3.7 A° (24° 2θ) corresponds to α-crystalline phase form in PA6. The α crystalline phase dominated the crystalline structure of prepared PA6. The line at 4.2 A° referred to α1 and originated from (200) plane whereas 3.7 A° originated from (002) plane^11^. The peaks observed at 11°, 22° and 23° can be identified as the γ-phase of the of PA6 with the corresponding indexes of (020), (001) and (200)/(201)^12^.

The number of unit cells (N) along their respective directions, define the broadening of the diffraction peaks. Though independent of the N, the peak area remains constant. Therefore, the peak broadening in XRD results was accompanied by decrease in the maximum peak height. However, as seen in Figure 3 (b) in main text the peaks were broadened, and intensity was decreased when silica functionalisation was employed on MNP.

There were many factors contributing to the observed peak profile. In essence, the peak profile shown in Figure 3 (b) in main text was a deconvolution of the peak from other contributions such as instrumental peak profile, crystallite size, microstrain, solid solution inhomogeneity, and temperature factors.

**Section 5 (S5): Full scale plots of induced magnetisation i.e. magnetic moment as a function of applied magnetic field, showing the associated hysteresis loops in inset figures at (a) 100 K and (b) 400 K temperatures.**

The SQUID characterisation results for the MNP samples are summarised as follows:





**(a)**





**(b)**

**Figure S4**. Full scale plots of induced magnetisation of MNPs (Magnetic moment as a function of applied magnetic field) and the associated hysteresis loops in inset figures at (a) 100 K and (b) 400 K temperatures.

**Table** **S4**: Summarised magnetic results at 100 K for uncoated MNPs and silica functionalised MNPs.

| Sample | Coercivity  (H_c_) (Oe) | Magnetic Remanence  (M_r_) (emu/g) | Magnetic Saturation  (M_s_) (emu/g) | Magnetic Moment Ratio  (M_r_/M_s_) (%) |
| --- | --- | --- | --- | --- |
| Uncoated-Fe_3_O_4_ MNPs | 238 | 3695 x 10^-4^ | 13210 x 10^-4^ | 28 ± 5 |
| TEOS-Fe_3_O_4_ MNPs | 224 | 287 x 10^-4^ | 839 x 10^-4^ | 34 ± 3 |
| Uncoated-SMNP MNPs | 54 | 493 x 10^-4^ | 7275 x 10^-4^ | 7 ± 6 |
| TEOS-SMNP MNPs | 261 | 873 x 10^-4^ | 11281 x 10^-4^ | 8 ± 3 |

**Table S5**: Summarised magnetic results at 400 K for uncoated MNPs and silica functionalised MNPs.

| Sample | Coercivity  (H_c_) (Oe) | Magnetic Remanence  (M_r_) (emu/g) | Magnetic Saturation  (M_s_) (emu/g) | Magnetic Moment Ratio  (M_r_/M_s_) (%) |
| --- | --- | --- | --- | --- |
| Uncoated-Fe_3_O_4_ MNPs | 82 | 2031 x 10^-4^ | 11853 x 10^-4^ | 17 ± 4 |
| TEOS- Fe_3_O_4_ MNPs | 81 | 1437 x 10^-4^ | 9527 x 10^-4^ | 15 ± 3 |
| Uncoated-SMNP MNPs | 29 | 69.17 x 10^-4^ | 4806 x 10^-4^ | 1 ± 5 |
| TEOS-SMNP MNPs | 22 | 32.95 x 10^-4^ | 3623 x 10^-4^ | 0.9 ± 4 |

The SQUID characterisation results for all the prepared nanocomposite samples are summarised as follows:


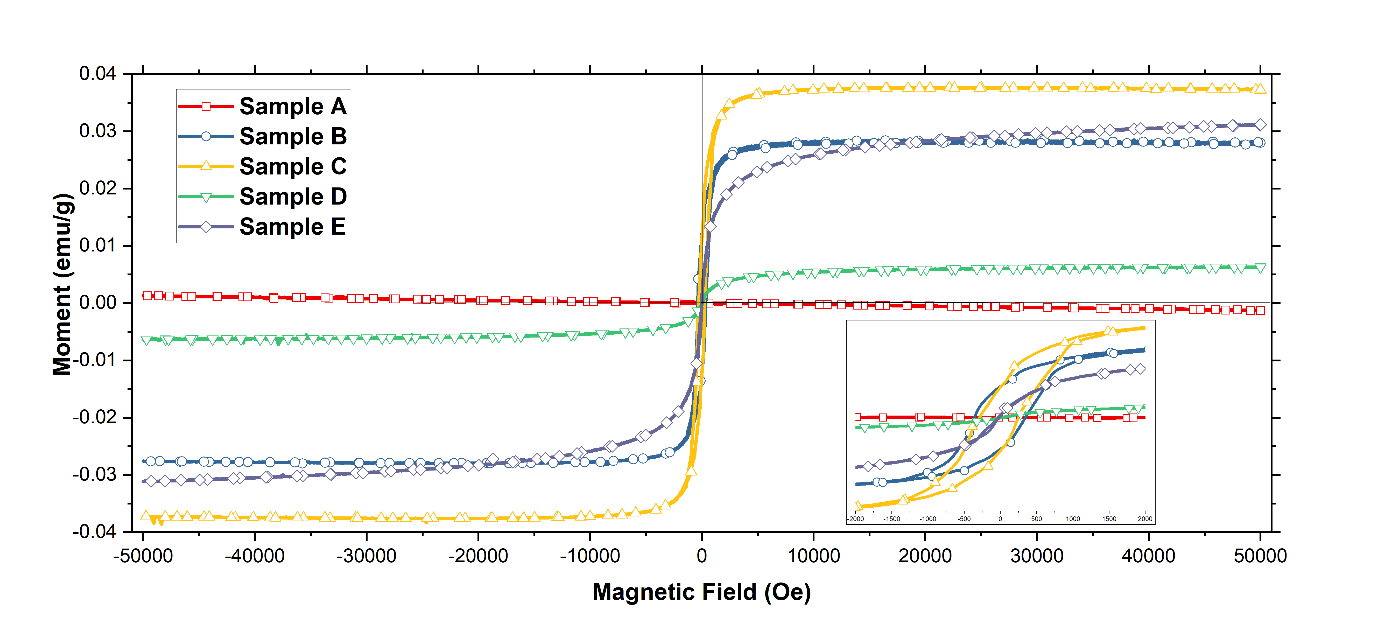


**(a)**


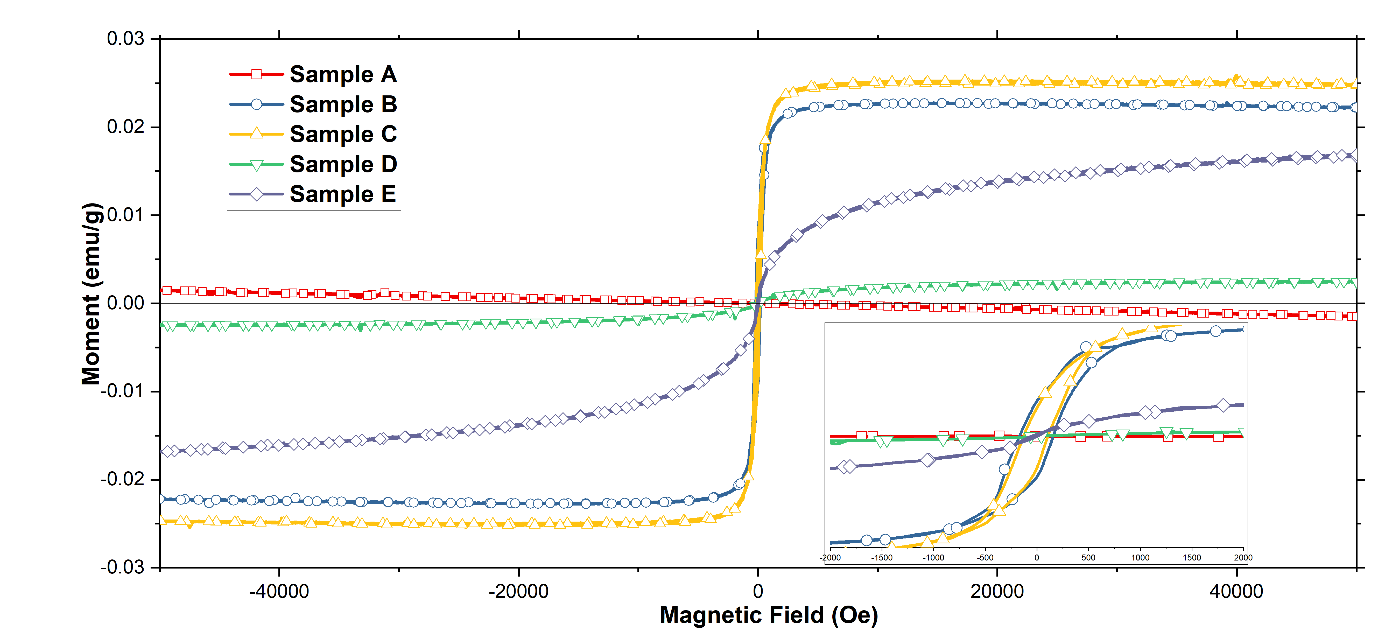


**(b)**

**Figure S5**. Full scale plots of induced magnetisation of PMC samples (Magnetic moment as a function of applied magnetic field) and the associated hysteresis loops in inset figures at (a) 100 K and (b) 400 K temperatures.

**Table S6**: Summarised magnetic results at 100 K for all the prepared samples.

| Sample | Coercivity  (H_c_)  (Oe) | Magnetic Remanence  (M_r_)  (emu/g) | Magnetic Saturation  (M_s_)  (emu/g) | Magnetic Moment Ratio  (M_r_/M_s_)  (%) |
| --- | --- | --- | --- | --- |
| *Sample A* | 0 | 0 | 0 | 0 |
| *Sample B* | 352 | 114 x 10^-4^ | 279 x 10^-4^ | 41 ± 4 |
| *Sample C* | 264 | 116 x 10^-4^ | 373 x 10^-4^ | 31 ± 3 |
| *Sample D* | 40 | 1.9 x 10^-4^ | 63 x 10^-4^ | 3 ± 4 |
| *Sample E* | 7 | 4.5 x 10^-4^ | 311 x 10^-4^ | 1 ± 5 |

**Table S7**: Summarised magnetic results at 400 K for all the prepared samples.

| Sample | Coercivity  (H_c_)  (Oe) | Magnetic Remanence  (M_r_)  (emu/g) | Magnetic Saturation  (M_s_)  (emu/g) | Magnetic Moment Ratio  (M_r_/M_s_)  (%) |
| --- | --- | --- | --- | --- |
| *Sample A* | 0 | 0 | 0 | 0 |
| *Sample B* | 162 | 75 x 10^-4^ | 224 x 10^-4^ | 33 ± 5 |
| *Sample C* | 117 | 62 x 10^-4^ | 247 x 10^-4^ | 25 ± 4 |
| *Sample D* | 39 | 5.3 x 10^-4^ | 27 x 10^-4^ | 20 ± 4 |
| *Sample E* | 31 | 4.6 x 10^-4^ | 166 x 10^-4^ | 3 ± 7 |

**Section 6 (S6): Original TEM micrographs used for the Photoshop editing and Simulated Model generation.**

The original TEM micrographs utilised for the analysing the agglomerates.


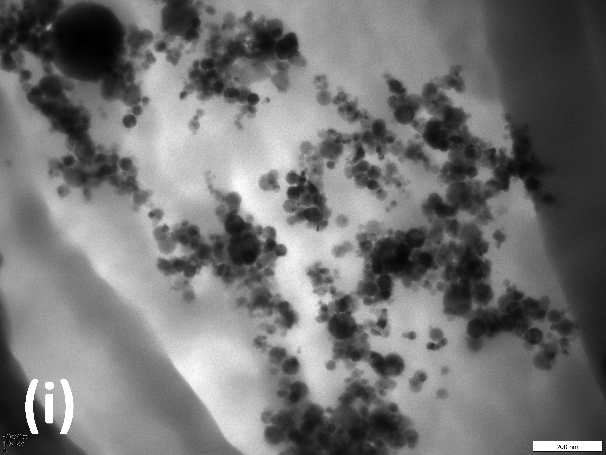

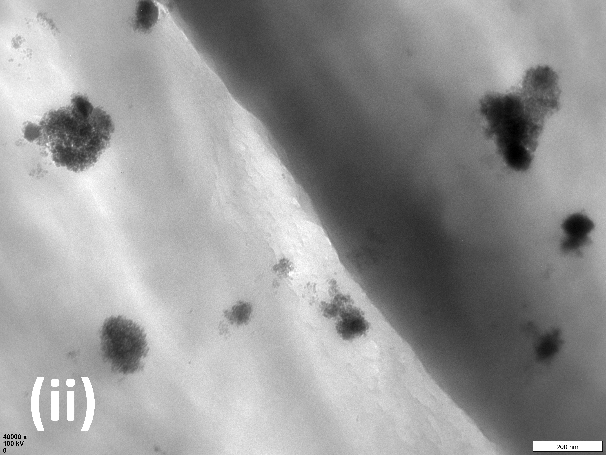


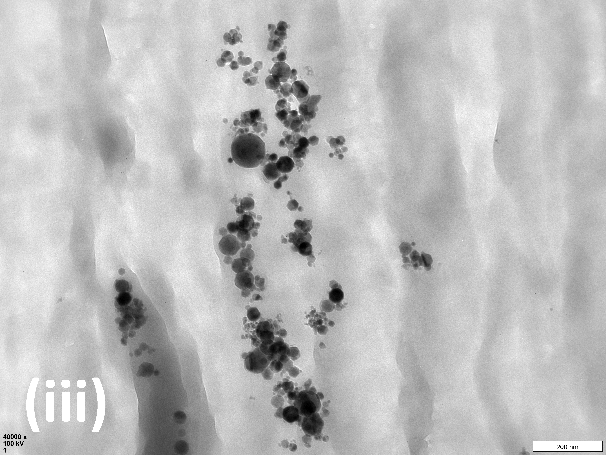

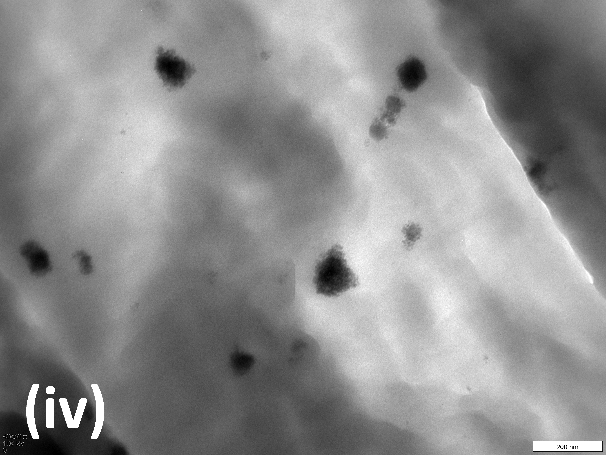


**Figure S6**: Original TEM micrograph images for uncoated samples (i) Sample B and (ii) Sample D; and coated samples (iii) Sample C and (iv) Sample E. (Scale bar shown is of 200 nm)

Considering all the characterisation data related with the dispersion state of the MNPs, the following summary table is prepared to be used as an input data in MATLAB^®^ code, to simulate the 3D model.

**Table S8**: Estimated diameters of nanoparticle/agglomerate regions identified from TEM, SAXS and XRD.

| Sample | TEM-Biggest agglomerate size (nm) | TEM-Smallest nanoparticle/ agglomerate size (nm) | SAXS calculation  of nanoparticle/ agglomerate size | XRD calculation  of nanoparticle/ agglomerate size |
| --- | --- | --- | --- | --- |
| sample B | 195 ± 10 | 25 ± 5 | 59 | 46 |
| sample C | 80 ± 15 | 30 ± 5 | 37 | 34 |
| sample D | 220 ± 20 | 50 ± 10 | 60 | 52 |
| sample E | 90 ± 10 | 40 ± 5 | 40 | 37 |

The simulated models representing the MNP/agglomerate arrangements in 3D are generated for all the samples as shown in Figure S7 below.


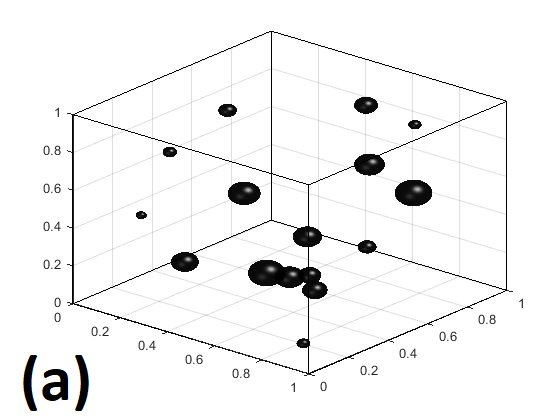

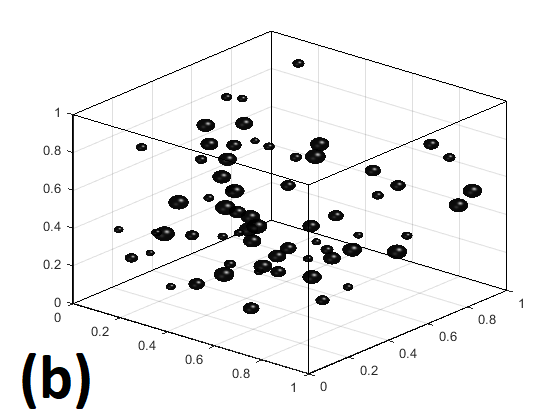


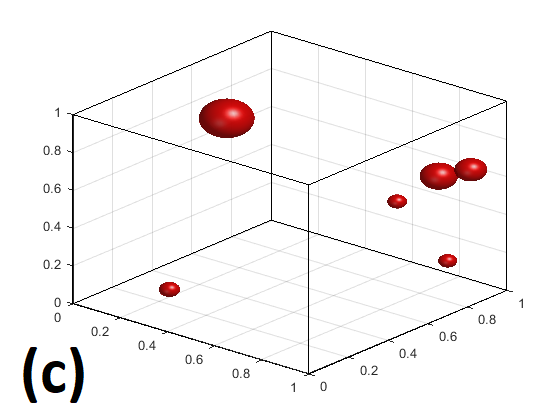

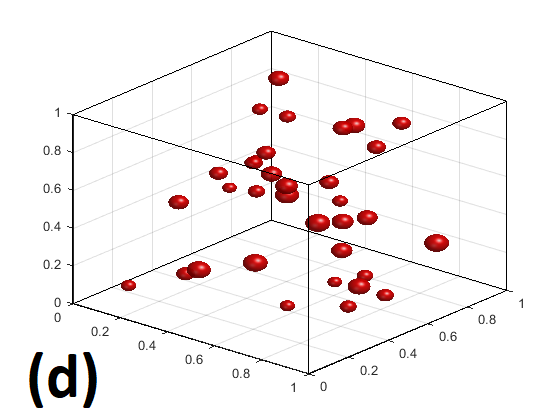


**Figure S7**. Simulated representation of the synthesised nanocomposite (1 cubic micron size) for Fe_3_O_4_ samples (a) Sample B, (b) Sample C and SMNP samples (c) Sample D, (d) Sample E respectively (Herein, Black spheres represent Fe_3_O_4,_ and Red spheres are for SMNP nanoparticle/agglomerate respectively.)

**References**

1. Darr, J. A., Zhang, J., Makwana, N. M. & Weng, X. Continuous hydrothermal synthesis of inorganic nanoparticles: applications and future directions. *Chem. Rev.* **117**, 11125-11238 (2017).

2. Gruar, R. I., Tighe, C. J., Southern, P., Pankhurst, Q. A. & Darr, J. A. A direct and continuous supercritical water process for the synthesis of surface-functionalized nanoparticles. *Ind Eng Chem Res* **54**, 7436-7451 (2015).

3. Campelj, S., Makovec, D. & Drofenik, M. Preparation and properties of water-based magnetic fluids. *Journal of Physics: Condensed Matter* **20**, 204101 (2008).

4. Iler, K. R. The chemistry of silica. *Solubility, Polymerization, Colloid and Surface Properties and Biochemistry of Silica* (1979).

5. Gupta, R. *et al*. Novel Method of Healing the Fibre Reinforced Thermoplastic Composite: A Potential Model for Offshore Applications. *Composites Communications* **16**, 67-78 (2019).

6. Hino, K. *et al*. Size distribution of gold nanoparticles covered with thiol-terminated cyanobiphenyl-type liquid crystal molecules studied with small-angle X-ray scattering and TEM. *Chemical Physics Letters* **460**, 173-177 (2008).

7. Piiadov, V., Ares de Araújo, E., Oliveira Neto, M., Craievich, A. F. & Polikarpov, I. SAXSMoW 2.0: Online calculator of the molecular weight of proteins in dilute solution from experimental SAXS data measured on a relative scale. *Protein Science* **28**, 454-463 (2019).

8. Blaine, R. *Determination of polymer crystallinity by DSC. TA Instruments* (2011).

9. Speakman, S. A. Estimating crystallite size using XRD. *MIT Center for Materials Science and Engineering*, 03-08 (2014).

10. Porter, R. Macromolecular physics, volume 3—crystal melting, Bernhard Wunderlich, 363 (1980).

11. Shete, P., Patil, R., Tiwale, B. & Pawar, S. Water dispersible oleic acid-coated Fe3O4 nanoparticles for biomedical applications. *J Magn Magn Mater* **377**, 406-410 (2015).

12. Khodabakhshi, K. Anionic polymarisation of caprolactam: an approach to optimising the polymerisation condition to be used in the jetting process. (2011).
